# Current status of digital health interventions in the health system in Burkina Faso

**DOI:** 10.1186/s12911-024-02574-4

**Published:** 2024-06-19

**Authors:** Bry Sylla, Boukary Ouedraogo, Salif Traore, Ousseni Ouedraogo, Léon Gueswendé Blaise Savadogo, Gayo Diallo

**Affiliations:** 1 Team AHead, Bordeaux Population Health INSERM-U1219, Univ. Bordeaux, Bordeaux, 33000 France; 2 Ministry of Health and Public Hygiene, Ouagadougou, Burkina Faso; 3 Public Health Team, Nazi Boni University, Bobo Dioulasso, Burkina Faso, France

**Keywords:** Digital health, Digital health intervention, Mapping, Health information system

## Abstract

**Background:**

Digital health is being used as an accelerator to improve the traditional healthcare system, aiding countries in achieving their sustainable development goals. Burkina Faso aims to harmonize its digital health interventions to guide its digital health strategy for the coming years. The current assessment represents upstream work to steer the development of this strategic plan.

**Methods:**

This was a quantitative, descriptive study conducted between September 2022 and April 2023. It involved a two-part survey: a self-administered questionnaire distributed to healthcare information managers in facilities, and direct interviews conducted with software developers. This was complemented by a documentary review of the country’s strategic and standards documents on digital transformation.

**Results:**

Burkina Faso possesses a relatively comprehensive collection of governance documents pertaining to digital transformation. The study identified a total of 35 digital health interventions. Analysis showed that 89% of funding originated from technical and financial partners as well as the private sector. While the use of open-source technologies for the development of the applications, software, or platforms used to implement these digital health interventions is well established (77%), there remains a deficiency in the integration of data from different platforms. Furthermore, the classification of digital health interventions revealed an uneven distribution between the different elements across domains: the health system, the classification of digital health interventions (DHI), and the subsystems of the National Health Information System (NHIS). Most digital health intervention projects are still in the pilot phase (66%), with isolated electronic patient record initiatives remaining incomplete. Within the public sector, these records typically take the form of electronic registers or isolated specialty records in a hospital. Within the private sector, tool implementation varies based on expressed needs. Challenges persist in adhering to interoperability norms and standards during tool design, with minimal utilization of the data generated by the implemented tools.

**Conclusion:**

This study provides an insightful overview of the digital health environment in Burkina Faso and highlights significant challenges regarding intervention strategies. The findings serve as a foundational resource for developing the digital health strategic plan. By addressing the identified shortcomings, this plan will provide a framework for guiding future digital health initiatives effectively.

**Supplementary Information:**

The online version contains supplementary material available at 10.1186/s12911-024-02574-4.

## Introduction

An effective healthcare system is essential for the overall well-being of a country, involving various resources and services that are focused on enhancing the health of the population [1]. Regardless of one’s socioeconomic status, healthcare systems must exhibit resilience and ensure fair and equal access to healthcare services. The World Health Organization (WHO) outlines six fundamental pillars that form the foundation of a healthcare system: leadership and governance, health information systems, healthcare services, supply of health products, health financing, and human resources [1, 2]. It is crucial to enhance these foundational elements to ensure the healthcare system operates effectively. This involves focusing on personnel, financial resources, information management, supplies, transportation, communication, and overall guidance [1, 2].

The health information system (HIS) is of great importance among these pillars because it plays a crucial role in enabling planning and coordination across all levels of the healthcare system [3]. An efficient HIS guarantees the presence of high-caliber, dependable data, which is essential for decision-making procedures and the efficient provision of healthcare [3]. Insufficient performance of the HIS can impede national planning endeavors, leading to missed chances to efficiently utilize resources and enhance health outcomes [4].

Recently, the incorporation of information and communication technologies (ICTs) has been identified as a possible solution to improve the resilience and effectiveness of healthcare systems [5]. Countries globally, especially in advanced regions such as Europe, the United States, and China, have initiated digital transformation efforts in their healthcare systems [6–9]. These endeavors have resulted in the implementation of electronic health records (EHRs), the sharing of medical records, the development of patient-centered applications, and the use of connected devices to improve the efficiency of healthcare delivery [8, 9] Nevertheless, there are still significant obstacles regarding data protection, data exchange, and ethical considerations that continue to be widespread [8].

The COVID-19 pandemic has expedited the implementation of digital health solutions in African nations, including Burkina Faso [10]. Although there has been progress, numerous digital health initiatives in developing nations suffer from a lack of clearly defined national strategies, which restricts their scope and impact [11–13].

Insufficient data collection and reporting in Burkina Faso have been recognized as obstacles to efficient healthcare management [14, 15]. However, it is crucial to note that DHIS2 was adopted in 2013 to facilitate the implementation of the national health data warehouse and a routine health services data management platform [16].

The objective of this research is to conduct a thorough analysis of digital health applications and solutions in Burkina Faso. The study aims to categorize and organize different interventions in order to provide insights for the establishment of a digital health strategic plan and to provide recommendations for the efficient deployment of digital tools for collecting primary data. By conducting this analysis, valuable information can be obtained to enhance Burkina Faso’s healthcare system and enhance the health outcomes of its population.

## Materials and methods

### Study design

**Fig. 1:**
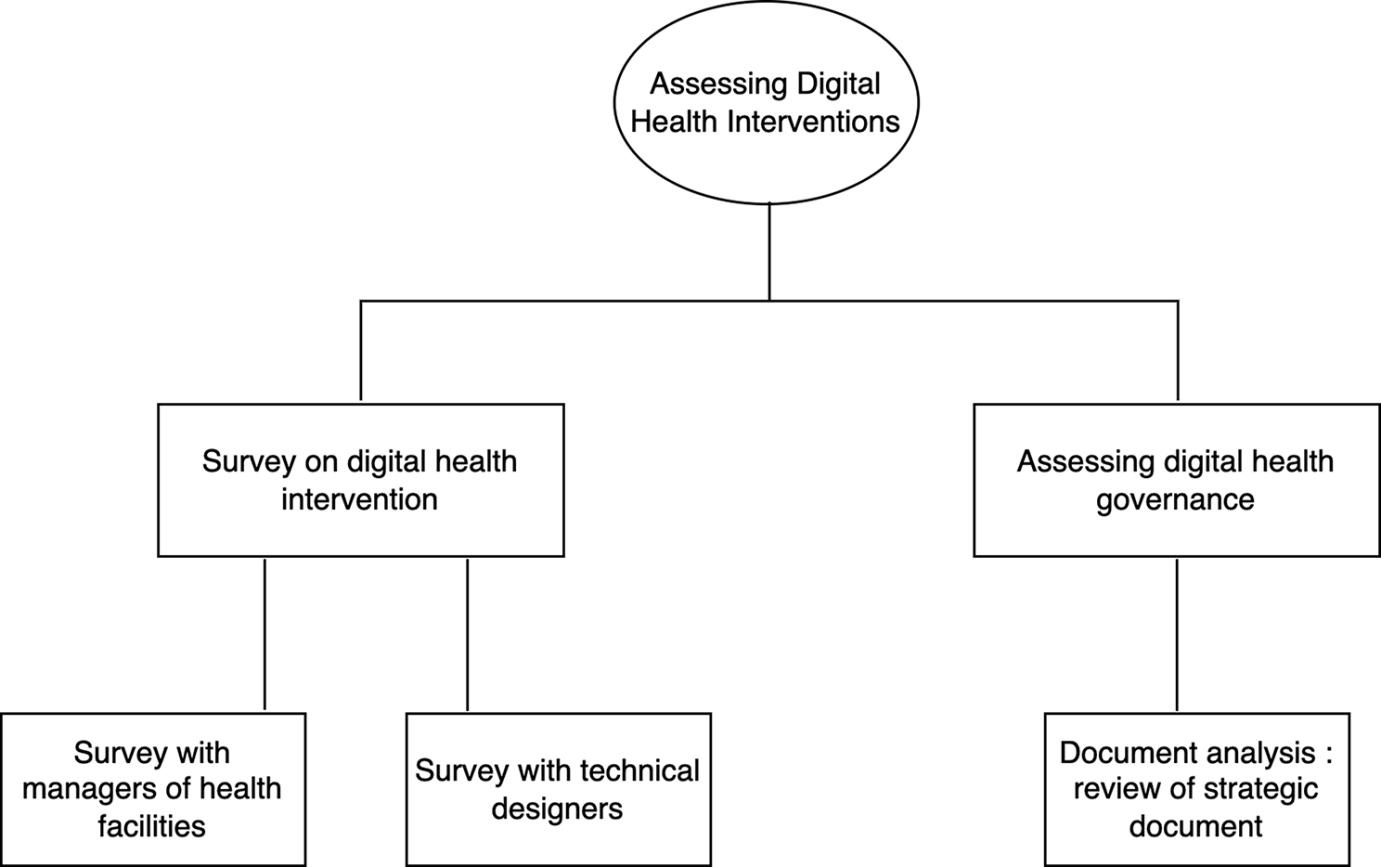
Flow diagram of study method

The study utilized a descriptive cross-sectional design with a retrospective approach to collect data, aiming to identify digital health interventions and assess strategic documents. It was conducted between September 2022 and April 2023 to identify the digital health interventions in Burkina Faso’s healthcare system and analyze the country’s digital health governance strategy from 2010 to 2022. This involved employing quantitative and document analysis methods, as illustrated in Fig. 1 of the overall study approach.

### Study population

The study population included public and private health facilities, as well as technical and financial partners (TFPs) such as the WHO, the Global Fund, the United States Agency for International Development, and the German Agency for International Development Cooperation, involved in developing and utilizing health system applications, software, and platforms. Burkina Faso’s healthcare system is structured in a pyramid with three levels:

- First level: health and social promotion centers (HSPCs) (*n* = 2,207), medical centers (*n* = 99), private medical practices (*n* = 617), medical centers with a surgical unit (MCSU) (*n* = 46), private clinics (*n* = 88).
- Second level: regional hospital centers (RHCs) (*n* = 9), private polyclinics (*n* = 2).
- Third level: university hospital centers (UHCs) (*n* = 6).

Designers included those identified during the first phase by surveyed healthcare structures as solution developers, mainly Ministry technicians, TFPs, and technicians from private health structures or IT solution companies.

### Sampling

To ensure comprehensive representation across various types of facilities nationwide, our sampling process was stratified into four distinct strata. Here’s a detailed breakdown of each stratum, ensuring the representativeness of the study population:

- Stratum One: This included RHCs, polyclinics, and UHCs, totaling 17 health establishments.
- Stratum Two: Encompassed MCSU and private clinics, totaling 134 health establishments.
- Stratum Three: Comprised of basic health facilities such as medical centers, private medical practices, HSPCs, and private medical practices, totaling 2,923 health facilities.
- Stratum Four: This stratum included designers, the exact number of which was initially unknown and was expected to correspond to the number of retained applications.

Our sampling approach involved a combination of probabilistic and non-probabilistic methods. Non-probabilistic sampling was employed based on careful observations of the digitization status of health structures in Burkina Faso, considering the potential for digitization in urban areas and the limited number of certain strata. Consequently, all structures within strata one and four, including hospitals and surveyed designers in phase two, as well as all urban facilities, were included due to their low numbers. On the other hand, probabilistic sampling was applied to the remaining structures in strata two and three that were not located in urban areas. In this approach, one-third (1/3) of these structures were randomly selected for inclusion in the study.

### Survey on digital health interventions

The survey on digital health interventions comprised two phases:

- Phase One: a survey with healthcare facility users, it addressed the overall description of the digital solutions, including application descriptions, designers, data management practices, funding sources, and classification according to the National Health Information System (NHIS), healthcare system, and user dimensions.
- Phase Two: a survey with technicians or Designers, this phase consisted of carrying out a more detailed survey focusing on technologies, standards, interoperability, administration, hosting, and the role of designers. Designers of digital health interventions, identified from the Ministry of Health’s list, were approached for insights.

two tailored questionnaires were devised: (i) the digital intervention form and (ii) the designer’s form. The data collection process entailed the completion of a self-administered questionnaire by the data management managers of the designated facilities, alongside face-to-face interviews with the designers of the identified applications. Subsequently, the collected data underwent analysis utilizing the R software.

To streamline the selection of applications or platforms and to facilitate comprehensive analysis, specific inclusion and exclusion criteria were delineated. These criteria are outlined in detail in Table 1.

**Table 1:** Inclusion and exclusion criteria

Inclusion criteria	Exclusion criteria
Digital intervention available and used by several users and structures within the healthcare system	Excels, Access base, billing and technical tray applications
Digital intervention developed by either the Ministry of Health or a private company and accessible in either public or private healthcare facilities	Personal applications designed for individual use

### Assessment of strategic documents

The document analysis employed to assess Digital Health Governance and leadership, and the architecture of the information system in Burkina Faso followed Sarah L. Dalglish et al.‘s READ (Review, Extract, Analyze, and Distill) approach, entailing the review of strategic documents related to digital health and the extraction of information to collect and analyze data systematically [17].

- Review: we established the criteria for the types of documents to be analyzed, focusing on strategic documents relevant to digital health governance and health information workflow. After a collaborative validation process, we scheduled time for search strategies to obtain relevant documents. This involved sourcing documents from the Ministry of Health’s Health Information Systems Department (HISD), reviewing the official websites of ministries and government institutions, and reaching out to key stakeholders to access unpublished documents.
- Extract data: data extraction involved carefully reviewing the selected strategic documents, including but not limited to The National Health Development Plan 2021–2030, The National Health Development Strategy 2020–2025, The National Health Information System Strategic Plan 2022–2025, The Hospital Information System Repository, and The National Strategy for the Development of the Digital Economy 2018–2027. We focused on extracting pertinent details regarding governance and leadership structures for digital health. This encompassed identifying the names of relevant bodies, their establishment dates, mandates concerning ICT integration in healthcare, and specifics regarding the architecture of the health information system.
- Analyze data: this analysis aimed to identify the strategy and areas covered by digital health governance in Burkina Faso, based on information extracted from strategic documents.
- Distil findings: The final step involved refining and summarizing the findings from the analysis. This included organizing the extracted data into categories related to digital health governance, creating visual representations to illustrate key findings, and presenting the results in a coherent narrative that addressed the research questions regarding digital health governance in Burkina Faso.

## Results

A comprehensive set of 972 forms was successfully completed following meticulous data processing and initial analysis aimed at identifying applications that were shared among multiple participants. A thorough review of applications and platforms across various tiers of the healthcare system yielded an extensive catalog of applications employed within the healthcare domain. A total of 221 applications and platforms were initially identified. However, after eliminating duplicate entries, the final count was reduced to 115 applications. The majority of both public and private facilities have already implemented billing management software, as well as software designed for the management of laboratory and radiography technical platforms. To facilitate a comprehensive analysis of the various domains of digital health intervention, certain software and applications, specifically billing and technical platforms, were excluded from consideration. Following the application of inclusion and exclusion criteria, a total of 35 applications and platforms were selected and subjected to analysis.

### The national health information system

**Fig. 2:**
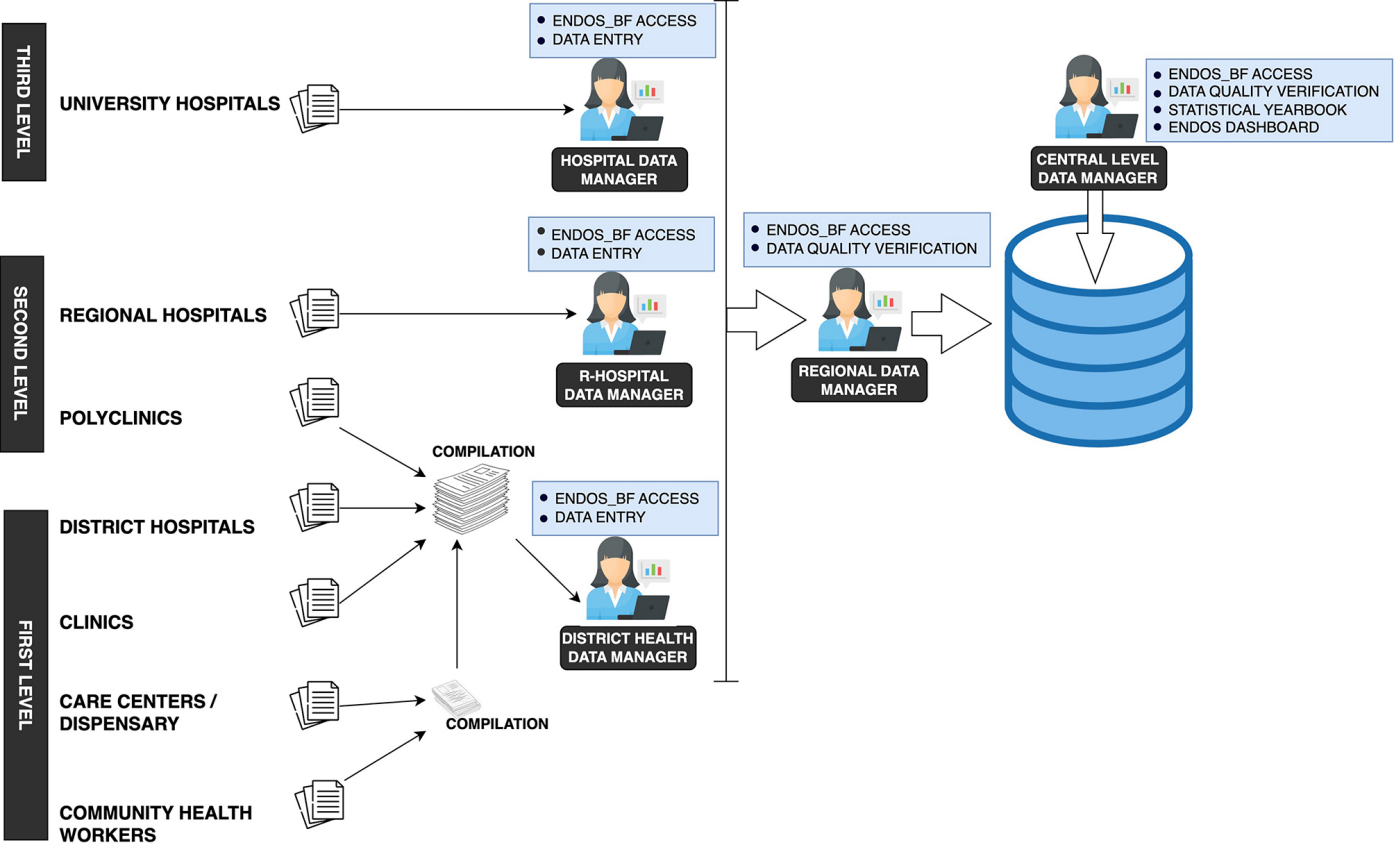
Routine health data collection architecture

In Burkina Faso, the predominant instruments employed for data collection in healthcare facilities consist of physical consultation registers. These registers serve as sources for recording information pertaining to consultations and various other activities. The aforementioned data are subsequently documented on a physical medium referred to as a “monthly activity report.” These reports are then transmitted to the district level, where data managers responsible for the respective regions enter them into the data warehouse. In the case of hospitals, the departments transmit the reports to the information manager, who then proceeds to enter them into the warehouse. Subsequently, the collected data are transmitted to the central level to undergo processes of validation, aggregation, analysis, and dissemination through the statistical yearbook. The flow of health information follows the different levels of the health system pyramid, the health information architecture is shown in Fig. 2.

### Governance and leadership

At the government level, Burkina Faso has established a Ministry of Digital Transformation, which is responsible for formulating, implementing and monitoring government policy on digital transition, postal services, and electronic communications in conjunction with the relevant ministerial departments. Created in 2002 to manage the postal, telecommunications, and IT sectors, it has since expanded its mandate in 2018 to encompass digital transformation [18].

The Data Protection Authority was established in 2004 in recognition of the need for a legal framework and the security of information systems. It has been operational since 2007, responsible for ensuring the protection of personal data. The National Information Systems Security Agency was created in 2013 to manage the security of Burkina Faso’s information systems and cyberspace.

To accelerate the digital transformation agenda at the national level, the National Agency for the Promotion of ICTs (NAPICT) was launched in 2014 to ensure the implementation of major development programs for ICTs. In line with this initiative, in 2016, the country set up a national data center, often referred to as the governmental cloud, under the auspices of the NAPICT, to host digitization projects within the country [18].

Additionally, in 2016, the government implemented legislation pertaining to standardizing the organization of ministerial departments (Decree No. 201 − 027/PRES/PM/SGGCM of 23 February 2016). This legislation officially established the inclusion of Information System Departments as an administrative entity within ministries and institutions.

HISD within the Ministry of Health was established in 2018 through Order No. 2018-0035/MS/CAB. Its primary responsibilities encompass the formulation of digital policies and strategies at the Ministry of Health and Public Hygiene (MSHP) level, as well as ensuring the consistency, security, and advancement of information systems in alignment with these policies and strategies. Additionally, the HISD is tasked with coordinating and overseeing the implementation of the Ministry’s digital e-health tools, as well as the national electronic patient file and hospital information system for hospital facilities. In summary, it assumes a leadership role in the implementation of digitization initiatives within the healthcare industry. This responsibility is carried out through collaborative efforts with various departments, organizations, partners, and the private sector [19].

### Financing mechanisms

Regarding the allocation of funds to ICTs in the healthcare sector through internal funding, it is noteworthy that the State budget does not include dedicated budgetary provisions for the advancement of digital health initiatives. However, the Ministry receives assistance from technical and financial partners in the realm of digitization. Nevertheless, the current resources available are inadequate to fully realize the aspirations for digital health [20].

The collected data offered a comprehensive representation of the proportion of funding allocated by technical and financial partners as well as the private sector, as presented in Table 2 below, which accounts for approximately 89% of the overall funding.

**Table 2:** Distribution of the contributory share of stakeholders in digital health

Source of financing	*N* = 35^1^
Ministry of Health	4 (11.43%)
Private sector	6 (17.14%)
Technical and financial partners	25 (71.43%)

^1^n (%)

### Overview of software applications

Despite the existence of explicit directives from leadership, the lack of sufficient internal financial resources has led to the establishment of parallel platforms. Recently, these measures have been executed in accordance with the specific demands of different projects and programs. In 2018, a comprehensive evaluation was conducted on various electronic databases utilized within the healthcare sector. This evaluation encompassed a wide array of databases, including Microsoft Excel and Access files, and unveiled the existence of approximately 80 databases [21].

### General information on applications

There was no specific nomenclature, and the names of the platforms were either linked to their designed purpose, such as *Base_Enquête, E_fluxfinancier, E_Certification*, or associated with fields of the NHIS or the specific pathology for which they were designed, such as *One Health, CPS-MILDA platform, cervical cancer platform*, or personalized names, such as *ENDOS, REC, STELAB.*

The implementation date of all the solutions falls within the period from 2012 to 2022, which proves that the last ten (10) years have been an active period of digital transformation in the healthcare system.

### Technologies used and categorization of digital health interventions

The inclusion of open-source software in national and regional ICT strategies was recommended in 2002 at a workshop organized by the Intergovernmental Agency of the Francophonie and the Economic Commission for Africa [22]. It is important to acknowledge that, as observed in Table 2, open-source technologies are primarily employed in the creation of applications, software or platforms used in digital health interventions, constituting 77% of the selected platforms, excluding the applications designed with Zend Framework 2 and unknown technologies. DHIS2 and Commcare, comprising approximately 54% of the selected applications, are utilized with high frequency. Approximately 20% of software utilized in the private sector is proprietary and is typically funded by the respective organizations themselves. Due to concerns regarding the safeguarding of intellectual property and the inherent skepticism among developers, the survey conducted did not gather specific details regarding the technological attributes of these proprietary solutions that were not collected in the survey, classified here as “unknown”.

It is worth noting that none of the applications has effective interoperability with the national data warehouse (Endos). According to the literature and as observed in Tables 3 and 60% (*n* = 35) of the technologies used have a possibility of interoperability with DHIS2. There is two-to-one interoperability between certain applications for certain specific needs.

**Table 3:** The different technologies used and their interoperability with Endos

Technologies used	Possibility of interoperability			
	**Unknown**, *N* = 8^1^	**No**, *N* = 6^1^	**Yes**, *N* = 21^1^	**Total**, *N* = 35^1^
CommCare	0 (0.00%)	0 (0.00%)	4 (19.05%)	4 (11.43%)
DHIS2	0 (0.00%)	0 (0.00%)	15 (71.43%)	15 (42.86%)
Unknown	7 (87.50%)	0 (0.00%)	0 (0.00%)	7 (20.00%)
Laravel	0 (0.00%)	1 (16.67%)	2 (9.52%)	3 (8.57%)
Maarch	0 (0.00%)	1 (16.67%)	0 (0.00%)	1 (2.86%)
Moodle	0 (0.00%)	3 (50.00%)	0 (0.00%)	3 (8.57%)
Xamp	0 (0.00%)	1 (16.67%)	0 (0.00%)	1 (2.86%)
zend_framework_2	1 (12.50%)	0 (0.00%)	0 (0.00%)	1 (2.86%)

^1^n (%)

**Fig. 3:**
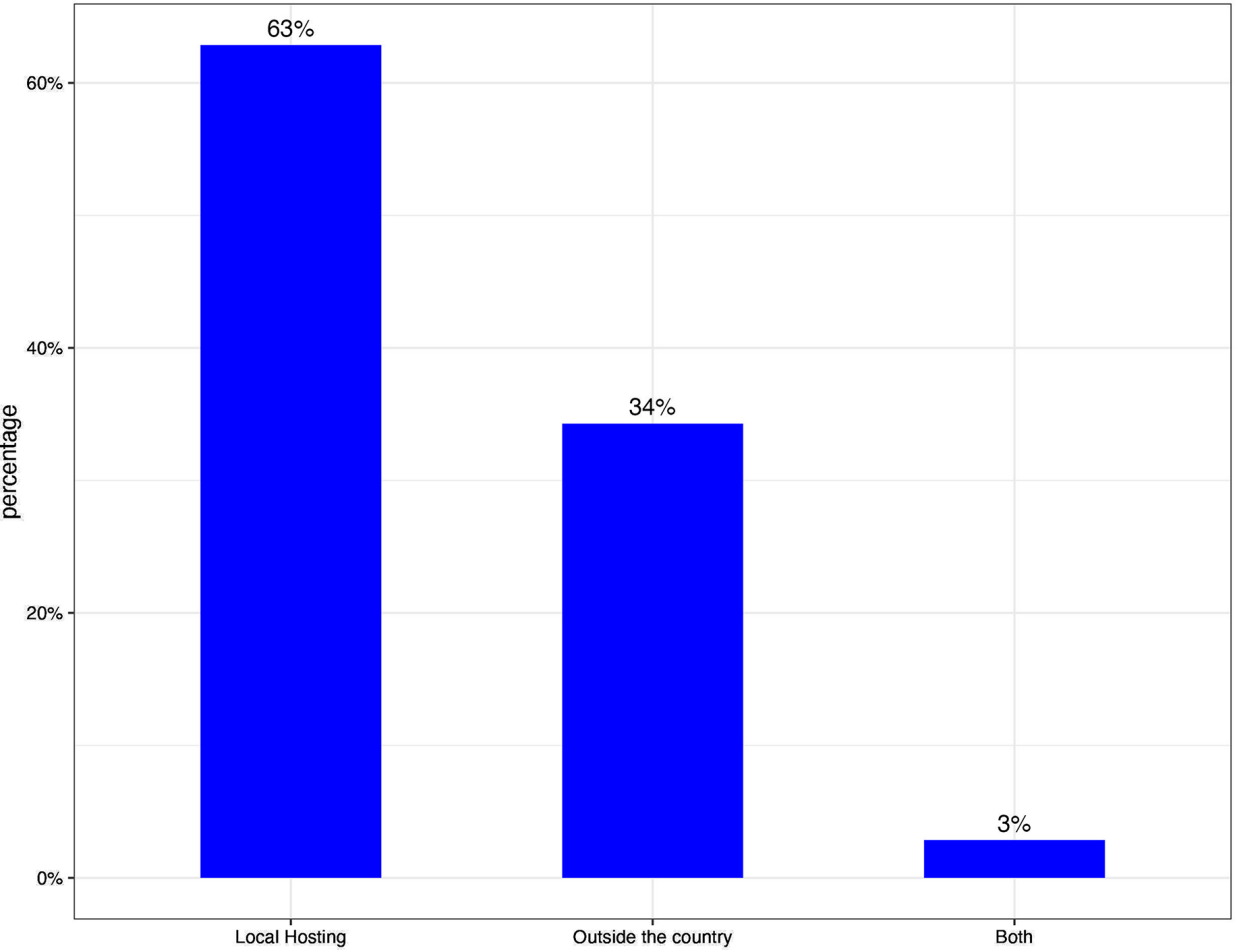
Distribution of platforms across the hosting locations

As previously indicated, since 2018, Burkina Faso has established a government data center, facilitating the hosting of various platforms on its national cloud infrastructure. Consequently, the study revealed that a majority of the software, specifically 22 instances accounting for 63%, is hosted within the country, while a smaller proportion of 12 instances, equivalent to 34%, is hosted outside the country. During the course of this study, three of the hosted platforms were undergoing migration to the cloud infrastructure within the country. The remaining platforms that are hosted beyond the national borders are predominantly utilized within the private sector. Out of the 22 platforms, 20 are currently hosted on the government cloud infrastructure, while the remaining 2 platforms are hosted on local physical servers within the country. One of the platforms exclusively employs four technologies in a progressive manner, with one of these technologies being hosted externally to the country. Figure 3 below shows the distribution of platforms according to their hosting locations.

The taxonomy of digital health interventions (DHI) defines the various modalities through which digital and mobile technologies are employed to address the requirements of the healthcare system [23]. It is a framework proposed by the WHO to classify health applications and software according to the health intervention area in which they are used. According to the main target users, digital health interventions are divided into the following categories:

- Clients: Clients are members of the public who are potential or actual recipients of health services, including health promotion activities. Caregivers of clients receiving health services are also included in this group.
- Healthcare providers: Healthcare professionals are members of the healthcare staff who provide healthcare services.
- Health System managers: Health system and resource managers are involved in the administration and supervision of public health systems. Interventions within this category illustrate management functions related to supply chain management, health financing, and human resources management.
- Data services: This is a cross-cutting functionality that supports a wide range of activities related to the collection, management, use, and exchange of data.

**Fig. 4:**
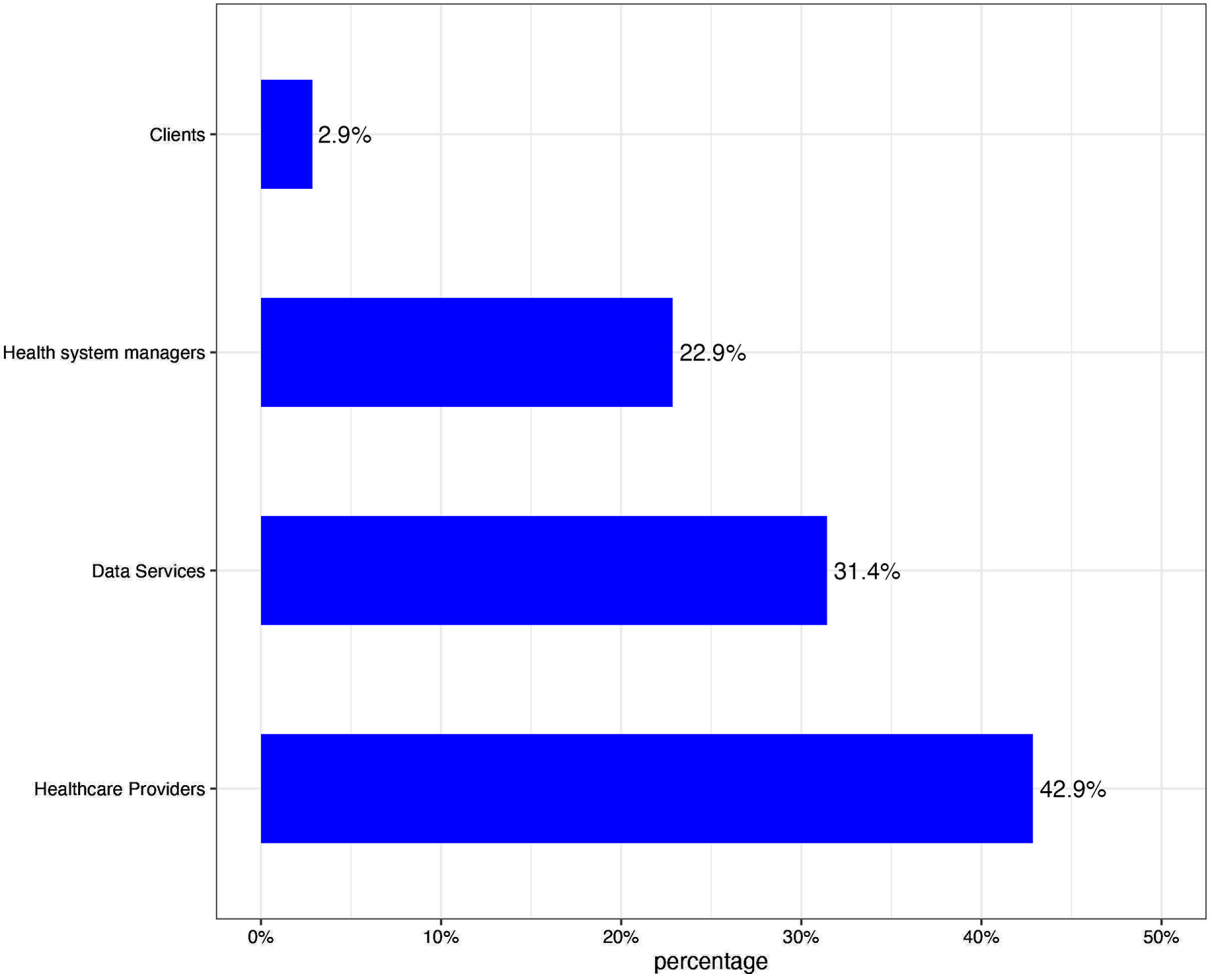
Categorization of applications across end users

Our study showed that digital health platforms are more focused on interventions for healthcare professionals (43%, *n* = 35) and data services (31%, *n* = 35), as observed in Fig. 4. Few applications are designed for patients (customers), and only one application was designed for customer use within the health system.

The 13 interventions utilized by healthcare professionals, with a sample size of 35, can be further categorized into sub-interventions. Among these, there are a total of nine (9) patient Electronic Health Records (EHRs), with six (6) originating from the public sector and three (3) from the private sector. Upon conducting an analysis, the following observations can be made:

- For the public sector, three (3) turned out to be electronic registers for consultations, two of which were tailored specifically to the pathologies of certain specific groups (children aged 0–5 and pregnant women), as well as and health programs. The remaining three (3) interventions comprised EHRs deployed in two university hospitals. Notably, two of these were specialty EHRs within the same structure, namely, the diabetes department and the orthopedics department. These EHR software packages only concern the consultation functional area and do not manage all the functionalities of the medico-administrative module. The third EHR software package is fully deployed in a university hospital and provides almost all the functionalities of the medico-administrative module of a patient file, including reception, consultation, hospitalization, movement and discharge. However, it does not include the cash/billing module, which is managed by another external application. It’s worth noting that both the Ministry of Health and the hospital’s technicians have limited knowledge regarding the technological features of the application. This limitation stems from the proprietary nature of the application, which was developed through a collaborative partnership between the country and China-Taiwan. The application employs the International Classification of Diseases 10th edition (ICD-10), while other codifications, such as procedures, have been tailored to align with the specific requirements of the local setting.
- For the private sector, these initiatives are privately funded and implemented in a modular manner, with the inclusion of different software modules varying depending on the specific structures involved. A comprehensive examination of the three applications, with a primary emphasis on technologies, standards, and norms, has uncovered challenges associated with insufficient information provided by the private entities responsible for designing the software. Among the three applications mentioned, two demonstrate notable utilization of ICD-10. The first application is the Inter Pharmaceutical Club, while the second application is VIDAL, which additionally incorporates both the French Common Classification of Medical Acts and the General Nomenclature of Professional Acts. It is important to acknowledge that the aforementioned applications are proprietary in nature and were procured by the clinics independently, without any prior engagement from the Ministry of Health and Public Hygiene.

**Fig. 5:**
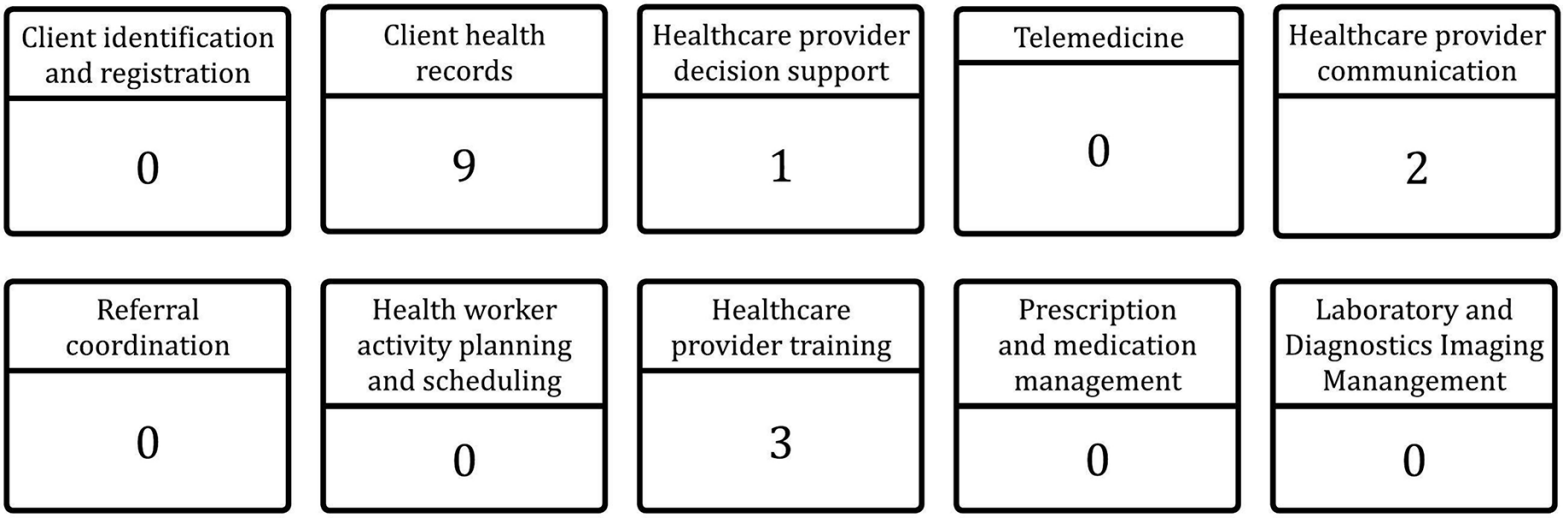
Distribution of digital health interventions aimed at healthcare professionals by sub-intervention

The lack of identification and telemedicine services was observed. It is worth mentioning that a telemedicine experiment was conducted in 2021; however, it did not progress to an operational stage. The distribution of interventions by sub-domain among healthcare professionals is depicted in Fig. 5:

A total of 31 digital health interventions were classified based on their respective contributions to enhancing the six pillars of the health system. The two pillars that have received the highest number of interventions are the health information system, which accounts for 32.2% of the interventions, and healthcare service delivery, which accounts for 35.4% of the interventions. Approximately 91% of digital health interventions in healthcare service delivery are currently in the pilot phase. Typically, digital health interventions are in the pilot phase in 61% of instances. The subsequent table presents a comprehensive breakdown of the health system pillars and the corresponding levels of implementation (Table 4).

**Table 4:** Distribution of digital health interventions according to health system strengthening priorities

Health system pillars	Stage of implementation		
	**On Scale**, *N* = 12^1^	**Pilot**, *N* = 19^1^	**Total**, *N* = 31^1^
Health system financing	2 (16.67%)	1 (5.26%)	3 (9.68%)
Leadership and governance	1 (8.33%)	1 (5.26%)	2 (6.45%)
Health workforce	2 (16.67%)	1 (5.26%)	3 (9.68%)
Medical products, vaccines and technologies	0 (0.00%)	2 (10.53%)	2 (6.45%)
Service delivery	1 (8.33%)	10 (52.63%)	11 (35.48%)
Health information systems	6 (50.00%)	4 (21.05%)	10 (32.26%)

^1^n (%)

The HIS of Burkina Faso is categorized into six distinct subsystems, namely, routine, epidemiological surveillance, program management, administration and resources, periodic surveys and studies, and the community subsystem [16]. Out of the 30 digital health interventions analyzed, 33% (*n* = 30) were primarily centered on the routine subsystem. Additionally, 30% of the digital health interventions are focused on the program management subsystem, specifically addressing drug logistics management, health financing, and the malaria program. Furthermore, 23% of the digital health interventions pertain to epidemiological surveillance. The distribution is outlined in Table 5.

**Table 5:** Distribution of digital health interventions by NHIS subsystems

NHIS subsystems	*N* = 30^1^
Administration and resource management	2 (6.67%)
Periodic surveys and studies	1 (3.33%)
Community health information system	1 (3.33%)
Program management	9 (30.00%)
Routine	10 (33.33%)
Epidemiological surveillance	7 (23.33%)

^1^n (%)

### The data

The platforms were categorized based on the nature of the data they collected, whereby platforms that collected individual data constituted 60% of the total, while platforms that collected aggregated data accounted for the remaining 40%. Among the platforms that gathered aggregated data, 58% (*n* = 14) are currently in the pilot phase. Similarly, for platforms that collected individual data, 71% (*n* = 21) are in the pilot phase. In total, the projects in the pilot phase accounted for 65.7% (*n* = 35).

The act of providing feedback was conceptualized as the creation of various written materials, such as newsletters, situation reports, and statistical yearbooks, which relied on the utilization of data derived from the applications. The utilization of data from digital health applications is observed to be low, as indicated by the provision of feedback in only 17% (*n* = 35) of the applications (Table 6).

**Table 6:** Interventions by data type and stage of implementation

Type of Data	Reuse of data		
	**No**, *N* = 29^1^	**Yes**, *N* = 6^1^	**Total**, *N* = 35^1^
Aggregate data	11 (37.93%)	3 (50.00%)	14 (40.00%)
Individual data	18 (62.07%)	3 (50.00%)	21 (60.00%)
**Total**, *N* = 35	29 (82,85%)	6 (17,14%)	35 (100%)

^1^n (%)

## Discussion

The present study enabled the analysis of digital health interventions and applications, software, or platforms used to implement these interventions within the Burkina Faso healthcare system, providing a comprehensive description of these applications in relation to their overall functionality, technological characteristics, and data-related aspects. Additionally, the categorization of the interventions was also conducted based on the WHO’s classification of digital health interventions, as well as the pillars of the healthcare system and the subsystems of the health information system. The results are elaborated upon in the subsequent section.

### Weak coordination of implementations

Digital technologies have a significant impact on enhancing healthcare accessibility, quality, and efficiency, particularly in the African context [24–26]. Nevertheless, numerous digital health initiatives encounter challenges in achieving long-term viability and seamless integration within healthcare systems. These setbacks can be attributed to a failure to account for the intricate sociotechnical systems within which they are implemented, as well as a dearth of effective coordination mechanisms [27]. Burkina Faso currently lacks a contemporary digital health strategic plan and documentation outlining interoperability standards and norms. Consequently, the existing digital applications within the healthcare system are numerous but implemented in a fragmented manner, failing to adequately address the areas requiring enhancement in the health system. Neumark et al. emphasized the significance of stakeholder collaboration involving government entities, the private sector, and non-governmental organizations to effectively implement and maintain digital health solutions [28]. The authors have further provided illustrative instances of successful digital health initiatives in the African region, such as the utilization of mobile health clinics and telemedicine services [28]. Based on the findings of our study, it was observed that all applications implemented within private sector 4 (*n* = 31), along with a limited number of applications implemented within public sector 4 (*n* = 31) by collaborating entities, remain undisclosed to the MSHP. The implementation of these applications occurred without adequate coordination or analysis of the prevailing circumstances among the different stakeholders, namely, the government, technical and financial partners, and the private sector.

According to Tambo E et al., there is a recognized requirement for political commitment and financial support from governmental entities and local stakeholders to fully realize the benefits of a digital healthcare system. In addition to the synchronization and uniformity of digital health strategies and methodologies, efforts are being made to enhance the provision of digital healthcare services at local, national, and regional scales [29]. However, the findings of our study indicate a comparatively limited involvement of local funding, accounting for only 11%, in digitization initiatives, in contrast to the substantial contribution of 89% from partners and the private sector. Certain platforms could potentially be integrated as modules within a unified platform. However, due to the diverse nature of the partners and funding sources involved, duplication may be necessary. Therefore, it is crucial to establish regulatory texts and strategic documents to provide guidance for digital health interventions.

### Open-source technology and insufficient use of standards in the digital environment

Open-source technology possesses the capacity to bring about a paradigm shift in the healthcare industry and enhance the well-being of a substantial number of individuals. By fostering a culture of collaboration, mitigating instances of corruption and implementing cost-reduction measures [30], open-source technology can bring about substantial benefits. This is particularly relevant for developing nations, wherein there is a growing inclination among private enterprises to develop healthcare software that lacks contextual adaptation. The findings of our study indicate that open-source technology is utilized in the development of applications in approximately 80% of cases. Various technologies currently exist that offer interoperability standards for computerizing the healthcare system. For instance, DHIS2 incorporates multiple APIs and adheres to the ADX standard. Additionally, there are predeveloped packages and external tools available, such as the ICD-10 exporter and a Python-based complementary development tool, which can be integrated to provide additional functionalities [31]. These options provide ample opportunities for exploring the computerization of the healthcare system.

One notable limitation that should be acknowledged is the lack of computerized patient records and comprehensive HISs within the public healthcare system. The limited number of initiatives that have been identified also demonstrates a lack of adherence to the norms and standards that are necessary for promoting interoperability. The considerable duration and substantial expenses associated with implementing a hospital information system prompt raise questions about the potential contribution of open-source software in the digitalization of primary data collection tools within healthcare facilities and hospitals. The significance of successful implementations of Electronic Health Records (EHRs) and HISs in developing nations is progressively gaining prominence within the healthcare sector [32, 33]. The study conducted by Campillo et al. highlights the positive impact of computerized records on enhancing the quality of care and facilitating data-driven decision-making. Specifically, their findings demonstrate that computerized records have led to improvements in the overall completeness of patient records while ensuring the maintenance of satisfactory clinical data quality [34].

### Shortcomings in digital health interventions

Collaborative application development facilitates the creation of adaptable platforms that can effectively cater to the diverse requirements of various stakeholders, such as patients, clinicians, and researchers. To effectively address evolving needs and technologies, it is imperative that digital tools possess the capacity for scalability and adaptability [35]. The increasing inclination to leverage ICTs in the healthcare sector has resulted in the disjointed advancement and execution of platforms and software, consequently leading to fragmentation. The analysis reveals that digital health projects and interventions exhibit a preference for isolated blocks, contingent upon whether the focus is on the pillars of the healthcare system, the end-users, or the subsystem of the health information system. Based on the pillars of the health system, it can be observed that digital health projects exhibit a preference for healthcare services (35.4%, *n* = 31) and the information system (32.2%, *n* = 31) at the expense of the remaining four pillars. In relation to end users, digital health interventions targeting healthcare professionals (43%, *n* = 31) and data services (31%, *n* = 31) were prioritized at the expense of the remaining two categories. About the subsystems of the country’s information system, digital health projects focused on routine subsystems (33%, *n* = 30), program management (30%, *n* = 30), and epidemiological surveillance (23%, *n* = 30) were found to have a detrimental impact on the remaining three subsystems. One significant obstacle that persists is the completion of digital health projects, as evidenced by a relatively low rate of 34% (*n* = 35) for projects that have been successfully finalized and implemented at a large scale.

Digital health interventions targeting professionals exhibit disparities in their distribution across various sub-domains. A higher number of patient record applications (9/13) were observed, primarily consisting of consultation registers or initiatives specific to a particular hospital specialty. The study revealed that computerized patient records in the private sector were adequately available. However, there was a noticeable absence of adherence to interoperability norms and standards. A single Health Information System (HIS) with adequate functionality was identified within a public hospital established through a collaborative effort between the host country and Taiwan, China. The absence of effective knowledge transfer to technicians within the Ministry of Health poses a significant barrier to the expansion, scalability, and replicability of the program in a different organizational setting. The utilization of hospital information systems is hindered by significant challenges associated with both environmental and human factors. Teaching hospitals present a greater level of complexity compared to nonteaching hospitals in this regard [36].

It is worrisome to observe that there are instances within the various groups (pillars, user groups, and subsystems) where interventions that could have been consolidated on a single platform are instead distributed, as well as applications that appear to share similar objectives. The aforementioned evidence highlights the limitations associated with the utilization of technologies in enhancing the healthcare system.

It is crucial to conduct a comprehensive evaluation of the implementation strategies employed in digital health initiatives and prioritize the completion of pilot projects that have demonstrated their capacity to enhance the healthcare system. This approach is preferable to initiating numerous pilot projects focused on digitalization that may remain in the pilot implementation phase. The presence of over 60% of interventions in the pilot phase serves as evidence of the wide distribution that characterizes the domain of digital public health.

### Data integration

In the existing body of literature, the concept of interoperability among various technologies has been acknowledged [31, 37]. However, our analysis revealed that there is a two-to-one interoperability link between certain DHIS2 databases for particular project requirements. Nevertheless, it is important to note that none of the individual collection platforms demonstrated effective interoperability with the national data warehouse for integration throughout the duration of the study. The establishment of a robust framework for the secure exchange and dissemination of healthcare data within a trusted setting is of utmost significance in facilitating the extensive adoption of digital health services [38].

### Limitations

Acknowledging the inherent limitations of our methodology, it’s crucial to note that our study relied on sampling techniques and self-administered questionnaires. Consequently, the reliability of our results may be affected by potential selection biases and the possibility of respondents skewing their answers to either exaggerate or conceal the truth.

Moreover, given the retrospective nature of our study, we did not assess users’ perceptions regarding the utilization of the applications, which represents a notable research perspective that warrants consideration.

## Conclusion

This study’s comprehensive assessment exposes critical shortcomings in Burkina Faso’s digital health landscape. Fragmented implementation, intervention duplication, and unequal distribution hinder progress. The absence of a national digital health strategy, interoperability framework, and robust health information system further impedes data-driven decision-making.

To propel Burkina Faso towards a robust digital health ecosystem, we recommend:

Developing a National Digital Health Strategy: This strategy should prioritize interventions that address identified gaps, promote coordinated implementation, and ensure equitable access across regions.

Establishing an Interoperability Framework: Seamless data exchange between healthcare facilities and digital health initiatives is crucial. A standardized framework will enable data aggregation and analysis to inform policy and improve service delivery.

Investing in Sustainable Solutions: A well-defined investment strategy should prioritize scalable and sustainable digital health solutions over unsustainable pilot projects.

Building Digital Infrastructure and Workforce Capacity: Continued investment in expanding mobile network access, fiber optics, and e-health/ICT training programs is essential.

Fostering User Engagement: Proactive user engagement strategies are crucial for technology adoption and maximizing the impact of digital health interventions.

By addressing these multifaceted challenges and capitalizing on advancements in mobile telephony and fiber optics, Burkina Faso can harness the transformative power of ICT to revolutionize healthcare delivery and optimize health outcomes for its citizens.

### Electronic supplementary material

Below is the link to the electronic supplementary material.

Supplementary Material 1

Supplementary Material 2

## Data Availability

The data obtained during the survey and analyzed in this current study are the property of Burkina Faso’s Ministry of Health and Public Hygiene. They are available from the corresponding author upon request.

## Abbreviations

DHIS2: District health information software version 2
HIS: Health information system
HISD: Health information system departement
HSPC: Health, social and promotion center
ICD-10: International classification of diseases version 10
ICT: Information and communication technologies
MCSU: Medical center with surgery unit
MSHP: Ministry of health and public hygiene
NAPICT: National Agency for the Promotion of ICTs
NHIS: National health information system
RHC: Regional hospital center
TFP: Technical and financial partner
UHC: University hospital center
